# Comment on “Indirect Fitness Benefits Enable the Spread of Host Genes Promoting Costly Transfer of Beneficial Plasmids”

**DOI:** 10.1371/journal.pbio.3001449

**Published:** 2021-12-21

**Authors:** Alastair Jamieson-Lane, Bernd Blasius

**Affiliations:** Institut für Chemie und Biologie des Meeres, Carl von Ossietzky Universität Oldenburg, Oldenburg, Lower Saxony, Germany

## Abstract

Plasmid transfer contributes significantly to bacterial evolution, but the forces selecting such generosity are poorly understood; this Formal Comment revisits a study which examined these forces both analytically and experimentally, making a correction to the algebra and reaching strikingly different results.

In their 2016 paper, Dimitriu and colleagues [1] make use of both experimental and analytical techniques to study horizontal gene transfer and the conditions under which indirect fitness effects select for cells with high donor ability. They report both empirically and theoretically that population bottlenecks can select for immobile genes that facilitate the spread of mobile gene elements. Unfortunately, while Dimitriu and colleagues’ in vitro and in silico work provides solid evidence of donor selection, the mathematical model used to explain these results does not agree with the conceptual model described in the surrounding paragraphs and displays nonphysical behavior. Here, we present a correction to the algebra, bringing it in line with the conceptual model presented; we find that selection for donor ability is no longer supported. We then discuss the key differences between Dimitriu and colleagues’ simulation (which select for plasmid donation) and their conceptual patch model (which does not), highlighting the subtle modeling decisions that lead the 2 models to diverge. Our sole focus in what follows is the patch model described in Dimitriu and colleagues’ paper (S1 Text [1]). We make no attempt to discuss other possible circumstances, such as preferential sharing, as explored elsewhere in their paper.

In their patch model, Dimitriu and colleagues consider a population of bacteria with plasmid transfer rate *q* and plasmid susceptibility *s*. Each bacteria either contains some focal plasmid (*p* = 1) or doesn’t (*p* = 0). At the start of each, cycle bacteria are split into an infinite number of patches, each with a small number of “founders” sampled from the global population. In each patch, bacteria multiply to large numbers, and then plasmid transfer takes place. The plasmid carriage, transfer rate, and plasmid susceptibility of founder *i* from patch *j* are denoted *p*_*i*,*j*_, *q*_*i*,*j*_, *s*_*i*,*j*_, respectively.

All founding strains are assumed to grow to equal numbers during the growth stage. Fitness effects apply during the colonization stage: Individuals have a base fitness *W*_0_, plasmid bearing individuals have an increased fitness *W*_0_ + *e*_*p*_, and conjugating individuals pay a cost *c*_*q*_*q*_*ij*_*p*_*ij*_. This cost is proportional to transfer rate and is paid only by those individuals carrying plasmids before the transmission stage. This cost is paid regardless of the presence of recipients.

Dimitriu and colleagues then assert that the fitness of individual *i* from patch *j* is the following:

$$W_{ij}=W_{0}+e_{p}\left[p_{ij}+\left(1-p_{ij}\right)p_{j}q_{j}s_{ij}\right]-p_{ij}c_{q}q_{ij},$$ (1)

with *p*_*j*_, *q*_*j*_ equal to the mean values of *p*_*ij*_, *q*_*ij*_ in patch *j*, calculated as $\sum \frac{p_{ij}}{n}$ and $\sum \frac{q_{ij}}{n}$, respectively.

While intuitively plausible, this *p*_*j*_, *q*_*j*_ term assertion runs into difficulties whenever *p* and *q* are correlated or when population numbers are low enough to create incidental correlations, as is the case during population bottlenecks—the particular case Dimitriu and colleagues wish to study. As a concrete example of this problem, consider a patch with 2 founding cells (see Fig 1). Cell A contains a plasmid, and cell B contains the machinery necessary to transport it.


$$p_{Aj}=1,q_{Aj}=0,s_{Aj}=1,$$

$$p_{Bj}=0,q_{Bj}=1,s_{Bj}=1.$$

**Fig 1:**
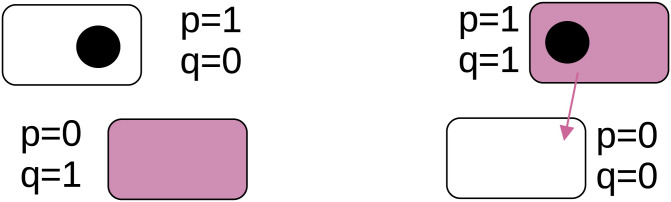
Simple example with 2 cells per cluster. Here, we use the same coding as Dimitriu and colleagues. Rectangles represent cells, circles denote plasmids, and red coloration indicates transfer apparatus. For both clusters illustrated, *p*_*j*_ = 1/2 = *q*_*j*_ and hence *p*_*j*_*q*_*j*_ = 1/4; however, in the right-hand case, conjugation is inevitable, while in the left-hand case, conjugation is impossible. [*pq*]_*j*_ distinguishes between these cases.

Naturally, nothing is going to happen, as the plasmid is stuck in a cell with no transfer apparatus. Nonetheless, *p*_*j*_ = 1/2 = *q*_*j*_ and hence *p*_*j*_*q*_*j*_ = 1/4, indicating a positive transfer rate in Eq 1. Eq 1 is thus inconsistent with the physical model described.

To determine plasmid availability in a population, it is not enough to determine the average occurrence of plasmids and transfer apparatus, but instead their average co-occurrence. Plasmid transfer takes place at a rate of (1 − *p*_*ij*_)*s_ij_*[*pq*]_*j*_, where ${\left[pq\right]}_{j}=\sum_{i}\frac{p_{ij}q_{ij}}{N}$, the mean of the product, as opposed to the product of the means. Under these assumptions, we have the following:

$$W_{i,j}=W_{0}+e_{p}\left[p_{ij}+\frac{s_{ij}}{N}(1-p_{ij})p_{ij}q_{ij}+\frac{s_{ij}}{N}(1-p_{ij})\sum_{i\neq k}p_{kj}q_{kj}\right]-p_{ij}c_{q}q_{ij}.$$ (2)

Here, we deliberately separate $\frac{s_{ij}}{N}(1-p_{ij})p_{ij}q_{ij}$ (the “self-transfer” term) from the rest of the sum. Because (1 − *p*_*ij*_)*p_ij_* = 0, we see that, inevitably, $\frac{s_{ij}}{N}(1-p_{ij})p_{ij}q_{ij}=0$. No cell can transfer plasmids to itself, nor to any “sister cells” that originate from the same founder. This is the critical difference brought about by [*pq*]_*j*_: While population bottlenecks increase relatedness in terms of *q* (making cooperation easier), they simultaneously increase relatedness for *p*, making plasmid transfer ineffective.

Assuming founding bacteria are sampled independently (as is done in Dimitriu and colleagues’ simulations), *p*_*kj*_*q*_*kj*_ will be independent of *q*_*ij*_, and we can calculate the regression coefficient *β*(*W*_*ij*_, *q*_*ij*_) as follows:

$$\beta \left(W_{ij},q_{ij}\right)=\frac{Cov\left(W_{ij},q_{ij}\right)}{Var\left(q_{ij}\right)}=\beta \left(s_{ij}\left(1-p_{ij}\right),q_{ij}\right)E\left(\frac{N-1}{N}q_{kj}p_{kj}\right)+\beta \left(e_{p}p_{ij},q_{ij}\right)-\beta \left(c_{q}p_{ij}q_{ij},q_{ij}\right).$$ (3)

If we also assume that *q*_*ij*_ is independent of *p*_*ij*_ and *s*_*ij*_ (a case Dimitriu and colleagues consider), this simplifies to

$$\beta \left(W_{ij},q_{ij}\right)=-E\left(p_{ij}\right)c_{q}.$$ (4)

The only possible effect from conjugation is negative, corresponding to the cost of conjugation. This contrasts with the corresponding result from Dimitriu and colleagues:

$$\beta \left(W_{ij},q_{ij}\right)=e_{p}E_{j}\left[\left(1-p_{j}\right)p_{j}\right]\beta \left(q_{j}s_{ij},q_{ij}\right)-p_{ij}c_{q}.$$ (5)

Dimitriu’s result suggests that *q* is favored if transfer is sufficiently efficient (*E*_*j*_[(1 − *p*_*j*_)*p*_*j*_] large); however, this possibility is an artifact of the *p*_*j*_*q*_*j*_ term. When considering the more appropriate [*pq*]_*j*_, the only transfer term that *q*_*ij*_ is multiplied by is the self-transfer term $\frac{s_{ij}}{N}(1-p_{ij})p_{ij}q_{ij}$, which is, by necessity, 0.

If we drop the independent genes assumption, Eq 3 indicates that *q* is selected for when either *β*(*e*_*p*_*p*_*ij*_, *q*_*ij*_) or *β*(*s*_*ij*_ (1 − *p*_*ij*_), *q*_*ij*_) compensate for the cost of conjugation; *q* must be sufficiently correlated with the beneficial *p* or *s* genes. As currently presented, the patch model does not provide any mechanism for producing this correlation. While it is possible to drop the assumption that founders are sampled independently, doing so would appear to be at odds with both the patch dispersal model described in Dimitriu and colleagues’ Supporting information (S1 Text [1]) and their simulation code (S1 Code).

Surprisingly, while Dimitriu and colleagues’ simulations do account for correlations between *p* and *q*, as described above, they nonetheless observe weak positive selection for *q*, contrary to Eq 4. This apparent contradiction can be resolved by examining the differences between the 2 models in more detail: The analytic model allows only a single “conjugation event” per transfer cycle, while Dimitriu and colleagues’ simulations allow conjugation across a 36-hour time window. In the simulations, a bacteria can both receive plasmids from an unrelated cell and subsequently donate plasmid to sister cell within a single transfer cycle (see Fig 2). This is not possible in the analytic model, as all conjugation takes place “simultaneously.” Hence, we see that donor selection in spatial models is a second-order effect: Fitness benefits rely on the occurrence of multiple consecutive conjugation events. This is suggestive of the importance of the “transcriptional burst after plasmid conjugation” [2], which has been observed experimentally, and is consistent with Dimitriu and colleagues’ observation in simulations that effect size was very small.

**Fig 2:**
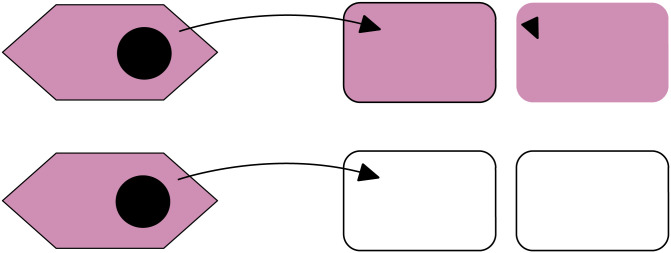
For transfer to be favored by purely spatial effects, a focal cell (center) must first receive a beneficial plasmid from an unrelated cell (left) and then transfer this newly received plasmid to a sister cell (right). This 2-step process is impossible when transfer takes place simultaneous and acts as a weak second-order effect favoring plasmid donors when serial conjugation is permitted (top row).

As shown by Dimitriu and colleagues, bacterial cooperation and spatial segregation can contribute to the evolution of plasmid transfer; however, as we show here, such results are sensitive to modeling details in particularly subtle ways. This leads to interesting questions about how robust such effects are in real life: Are population bottlenecks and spatial structures a significant selective force in the selection and maintenance of plasmid conjugation? Or does the sensitivity of the models reflect fragility in the real-world phenomena? These questions we leave to future authors.

## Supporting information

S1 Code Simulation code from Dimitriu and colleagues’ paper. Zip file containing the MATLAB code originally used by Dimitriu and colleagues, included here for the sake of model comparison, with permission of the original authors.
(ZIP)
