# Supplementary figures and images for: Comment on “Indirect Fitness Benefits Enable the Spread of Host Genes Promoting Costly Transfer of Beneficial Plasmids”
Source: PLoS Biol. 2021 Dec 21;19(12):e3001449. doi: 10.1371/journal.pbio.3001449 (PMC8691605; doi:10.1371/journal.pbio.3001449)

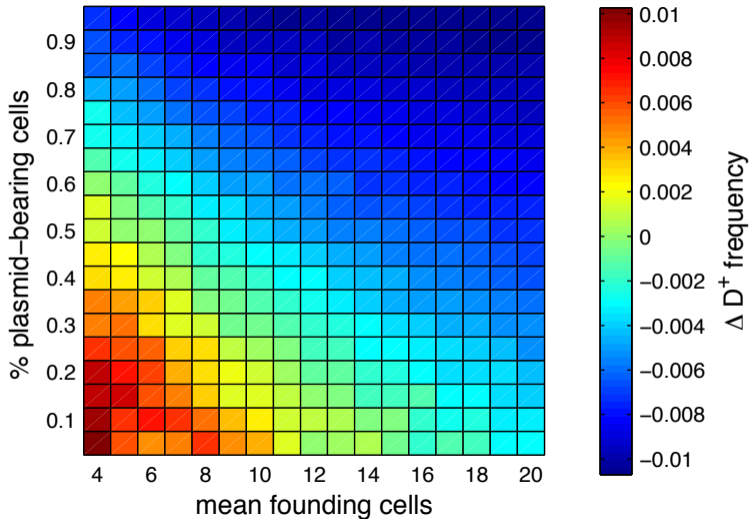

Supplement: S1 Code — Zip file containing the MATLAB code originally used by Dimitriu and colleagues, included here for the sake of model comparison, with permission of the original authors. (ZIP) [file pbio.3001449.s001.zip › poisson/fig4.pdf]

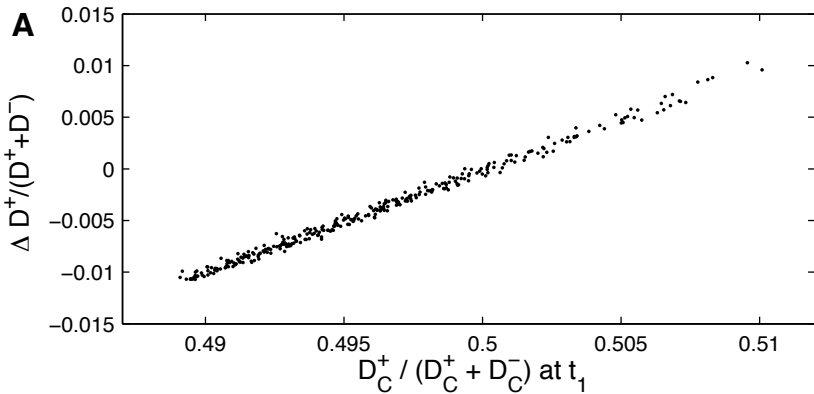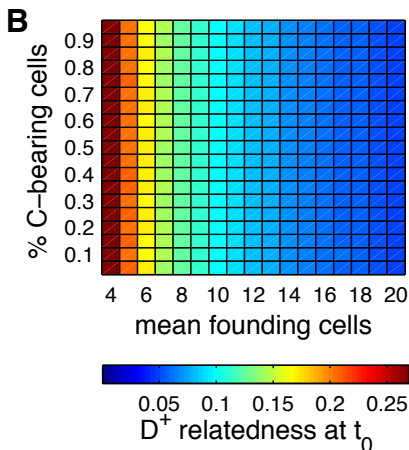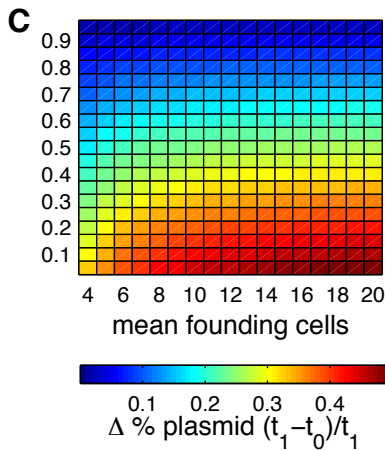

Supplement: S1 Code — Zip file containing the MATLAB code originally used by Dimitriu and colleagues, included here for the sake of model comparison, with permission of the original authors. (ZIP) [file pbio.3001449.s001.zip › poisson/figS5.pdf]
